# Investigating the Link between Eating Attitudes, Taste and Odour Preferences and the Chemical Senses

**DOI:** 10.3390/biology12111415

**Published:** 2023-11-10

**Authors:** Layla St Clair, Alyssa Grady, Mehmet K. Mahmut

**Affiliations:** Food, Flavour and Fragrance Lab, School of Psychological Sciences, Macquarie University, Sydney, NSW 2109, Australia

**Keywords:** disordered eating, anorexia nervosa, bulimia nervosa, olfaction, gustation, food preferences

## Abstract

**Simple Summary:**

Women with clinically diagnosed eating disorders have shown changes in their ability to taste and smell. However, whether this is true for non-clinical populations is unknown. We conducted the first study investigating whether high levels of disordered eating attitudes were associated with poorer taste and smell ability in a non-clinical sample. Our results indicated higher levels of disordered eating attitudes may be associated with different sensitivities to the odours from sweet and fatty foods compared to those with lower levels of disordered eating attitudes. However, further research is required to confirm our findings.

**Abstract:**

Objectives: To examine if higher degrees of pathological eating attitudes in a non-clinical sample are associated with odour and taste perception and preferences based on psychophysical ratings. Participants and Methods: A total of 80 female university students completed the eating attitudes test (EAT-26), followed by four chemosensory measures including olfactory and gustatory perception plus perceptual ratings and preferences for food odours and tastes. Results: There were no significant correlations between EAT-26 scores and measures of olfactory and gustatory perception. However, a significant interaction effect indicated higher degrees of pathological eating attitudes may be associated with differential sensitivity to sweet and fatty food odours compared to those with lower levels of pathological eating attitudes. Conclusions: This was the first study to examine pathological eating attitudes using food stimuli with a non-clinical sample. The results remain preliminary until replication. However, the findings highlight the need for development of measures of disordered eating attitudes and behaviours that go beyond caloric restriction.

## 1. Introduction

Eating disorders (EDs) are one of the most devastating health problems amongst young females worldwide [1], with anorexia nervosa (AN) having the highest mortality rate of any psychiatric illness [2,3]. Nevertheless, the mechanisms underlying the aetiology, symptomology, and maintenance of EDs remain poorly understood. Previous research has suggested that individuals with EDs, including AN, have an impaired sense of smell and taste [4,5]. Such research may have important implications for the treatment of EDs [6,7], as smell and taste are crucial determinants of food preference, selection, and consumption [8].

### 1.1. Pathological Eating and Olfactory Dysfunction

Olfactory dysfunction is associated with numerous psychiatric conditions, including schizophrenia [9,10], depression [11] and bipolar disorder [9] (BD). The link between poor odour identification and EDs was first explored by Kopala et al. [12] employing the University of Pennsylvania smell identification test [13] (UPSIT) with hospitalized female AN patients diagnosed using the Diagnostic and Statistical Manual (DSM) criteria and female healthy controls (HCs). Findings indicated that patients with AN had intact olfactory function, on par with HC’s, despite abnormal BMIs [12]. In a non-clinical study using the eating attitudes test (EAT-26) as a measure of eating pathology and one bitter tastant only, Stafford and colleagues [5] found that higher levels of eating pathology were associated with poorer acuity for the bitter tastant and poorer odour acuity [5].

Using a more complete measure of olfactory function, Fedoroff et al. [14] employed the olfactory threshold sensitivity test, a measure of odour acuity, as well as the UPSIT, to a sample of 55 females who had either AN, BN (bulimia nervosa), both AN and BN or were HCs. Results indicated that patients with AN displayed significant olfactory impairment across both olfactory function tests, which did not improve following weight gain. Using the Sniffin’ Sticks measure of olfactory function with a sample of female AN patients and female HCs, Roessner et al. [7] found that AN patients had an intact odour identification ability, but displayed moderate odour discrimination deficits and pronounced odour threshold deficits. However, in contrast, a similar study found a small (but significant) difference between AN patients and HCs on the Sniffin’ Sticks [15] odour identification test [16]. In addition, Bentz et al. [17] found that both first episode and weight-recovered AN patients displayed heightened odour threshold and identification ability when compared to HCs. These inconsistent findings suggest that other factors, including biological and cognitive differences, drive the olfactory dysfunction displayed by ED patients, rather than the extreme caloric restriction alone, which is explored in the following sections.

### 1.2. Pathological Eating and Gustatory Dysfunction

Gustatory dysfunction is associated with both cognitive and psychophysical impairments. For example, in a large study with older adults (*n* = 1376), Churnin et al. [18] found that gustatory dysfunction was associated with poorer performance on delayed word recall task. Moreover, long-term malnutrition and biochemical abnormalities due to starvation-related disordered eating has been linked to gustatory dysfunction, specifically, hypogeusia (reduced sensitivity to taste) and dysgeusia (unpleasant perception of taste), resulting in food refusal and reduced appetite [19,20]. Clinical behavioural observations made in DSM-diagnosed AN patients, such as the use of high levels of salt, pepper and artificial sweeteners in meals, has been in part attributed to gustatory dysfunction [21,22].

In a study by Nozoe et al. [23], taste recognition across the four basic tastes (sweet, salty, sour, and bitter) was found to be significantly lower in hospitalized AN patients (*n* = 9) than in HCs (*n* = 6) at the commencement of treatment. Note that the diagnosis of anorexia was based on a Japanese Ministry of Health and Welfare [23]. However, once calorie intake reached 1600 Kcal/day, taste scores significantly improved. Notably, sensitivity to sour and bitter tastes rapidly improved after a daily intake of 1600 Kcal, while improvement to sweet and salty taste sensitivity occurred just prior to discharge. In another study, Frank et al. [24] found that both DSM-classified AN and obesity were associated with reduced taste classification accuracy for a sucrose solution, indicating food deprivation and overstimulation may be responsible for gustatory dysfunction.

However, the finding that ED individuals display reduced sensitivity to gustatory stimuli has not been consistently replicated. For example, in a self-reported measure of taste sensitivity, individuals with AN reported a hyper-sensitivity to taste stimuli, rather than reduced gustatory function [25]. Moreover, another study found that AN and BN patients did not differ from HCs in their estimates of the sweetness and fattiness of different stimuli [26]. In contrast, another study found that females with AN incorrectly assessed the taste of sucrose more often than female HCs, conflating sweet stimuli as bitter tasting [27]. Taken together, current research suggests that gustatory function and sensitivity varies across the spectrum of eating pathology, but that these deficits are reversable with treatment and weight regulation [4,20].

### 1.3. Pathological Eating and Odour/Taste Preferences

Smell and taste play a key role in shaping how pleasurable individuals find foods, with sweet sensations typically regarded as pleasant, and bitter sensations as unpleasant [28,29]. However, in populations with high pathological eating attitudes, food preferences and attitudes are largely shaped by psychological factors such as anxiety around weight gain and caloric intake, rather than by sensory experience alone [30]. For example, individuals with high pathological eating attitudes report liking only those foods which they viewed as nutritious [31]. This finding was replicated in a study with a non-clinical sample employing the EAT-26 where 25% of the sample met the cut-off criteria for an ED and reported restrictive eating health reasons [32]. Moreover, a review highlighted that patients with AN often report an aversion to fatty and sweet-tasting foods, perceiving them as highly caloric and eliciting their fear of weight gain [33].

One of the key components of AN is a general reduced pleasure derived from eating [31]. Several studies have explored the hedonic response to odour and taste stimuli in ED populations. For example, Simon et al. [34] found that DSM-classified AN patients have a reduced hedonic response when confronted with a typically pleasant taste stimuli i.e., high sucrose solution. In contrast, a review by Keating et al. [35] reported that hedonic properties of taste stimuli remain intact in AN patients, but the motivation for the stimuli was decreased, subsequently leading to reduced preference driven by a fear of weight gain. Most findings suggest that AN patients prefer highly sweet stimuli [26,36], while disliking highly fatty stimuli [31], with Sunday and Halmi [37] finding that DSM-classified AN patients displayed an aversion to all tastant stimuli that did not contain sugar.

### 1.4. Limitations of Previous Research

Due to varying methodological approaches, diverse assessment procedures, and failure to control for potentially confounding variables such as hunger level and BMI, the findings of previous studies have been mixed. Although clinical studies suggest that individuals with AN have an impaired sense of smell and taste, these findings cannot be generalized to the non-clinical population due to lack of research. Moreover, no studies to date have utilized real food as stimuli to investigate eating attitudes and tests of olfactory ability and taste perception with a non-clinical sample, and thus it is unclear whether previous findings extend to this population.

### 1.5. The Present Study Aims and Hypotheses

The aim of the current study was to examine if higher degrees of pathological eating attitudes in a non-clinical sample are associated with smell and taste deficits and preferences. It is hypothesized that (1) higher levels of disordered eating attitudes will be associated with lower olfactory ability; (2) higher degrees of disordered eating attitudes will be associated with a higher perceived pleasantness of high sugar, healthy food odours; (3) higher levels of disordered eating attitudes will be associated with lower psychophysical ratings (i.e., sweetness, bitterness, fattiness, intensity) of taste stimuli and (4) higher degrees of disordered eating attitudes will be associated with a higher perceived pleasantness of sweet tastes.

## 2. Method

### 2.1. Participants

Eighty female university students participated in the study in exchange for course credit. The participants were female only to allow comparison to past literature. Participants who had food allergies or cold/flu-like symptoms were asked not to participate. Participants were recruited via two parallel routes. One method of recruitment was an advertisement on an online recruitment platform which lists studies that students can self-enroll into. Due to the low incidence rate of high pathological eating attitudes, the second recruitment method involved inviting participants to complete a series of screener surveys, including the EAT-12 [38]. Participants with high scores on the EAT-12, indicating potential disordered eating attitudes, were invited to participate in the study. The use of these two recruitment methods was employed to ensure a representative distribution of participants with EAT-12 scores. The participants were aged between 17 and 53 years (*M* = 19.65 years, *SD* = 6.14). G*Power software (v. 3.1.9.7) was used to calculate the minimum sample size required for a medium effect size (*d* = 0.40) with 80% power, which was 50. Macquarie University Ethics Committee approved the ethical aspects of this study (Ref. 52022951936643).

### 2.2. Measures

#### 2.2.1. Hunger

Hunger was measured via the question “How hungry do you feel right now” presented on a sheet of paper. Participants were asked to circle the number on a 10-point scale that applied to their current state of hunger (1 = ‘not at all’, 10 = ‘extremely’). Hunger was measured four times throughout the experiment, and a mean score was calculated for the four individual hunger scores.

#### 2.2.2. Health Questionnaire

Health status relating to olfaction and gustation was measured via five questions, designed to screen-out those with past or present conditions/injuries that may compromise their sense of smell or taste. Participants were asked to disclose any allergies, medical conditions, or operations, as well as their use of cigarettes/vapes and potential exposure to chemicals, dusts or gases. No participants were excluded based on the results of this questionnaire.

#### 2.2.3. Eating Attitudes Test (EAT-26)

The EAT-26 [39] is a widely used self-report measure of disordered eating behaviours and attitudes in both clinical and non-clinical populations. The EAT-26 contains 26 items with questions on dieting, bulimia, and food preoccupation (e.g., “I feel extremely guilty after eating”) and response options “always” (3), “usually” (2), “often” (1), “sometimes”, “rarely” and “never” (each scoring 0). After reverse scoring one item, the total score is calculated by summing the scores of each response. Scores range from zero to 78, and scores of 20 or more are considered clinically significant. The test–retest reliability of this measure is adequate (r = 0.82 − 0.90, *p* < 0.001) [40]. Acceptable internal consistency was also demonstrated upon inspection of Cronbach’s alpha (α = 0.88).

#### 2.2.4. Olfactory Function (Threshold and Discrimination)

Olfactory function was measured using two tests from the Sniffin’ Sticks odour threshold and odour discrimination [15]. The tests are administered using felt tip pens (Burghart Instruments, Hamburg, Germany) that are held at approximately 2 cm in front of the participant’s nostrils while they are blindfolded, to avoid visual identification of the correct pen. The pens are presented with an interval of two seconds between pens, and 30 s between two triplets. For the threshold test, the test target pen contains n-butanol in 16 different dilutions of increasing strength (with pen level 1 being the strongest). The pens are presented in a staircase method, where stimulus concentration is decreased following trials that elicit a correct response and increased following trials that elicit an incorrect response. The average of the last four up–down transitions (reversals) is the participant’s threshold sensitivity score (ranging from 1–16, with higher scores representing better olfactory sensitivity).

The odour discrimination test involves the presentation of 16 different triplets of pens where two contain the same odour, and one contains a different odour (the target). Once again, participants are presented with three pens at a time, and asked to identify the target pen in a triple forced-choice paradigm. The stimuli are common odours including foods and flowers such as lemon, cloves, rose and orange. The discrimination score is calculated by the total number of correct trials out of 16 (ranging from 0–16), with higher scores representing greater olfactory discrimination ability.

#### 2.2.5. Food Odour Rating Task

Odour sensitivity and preference was measured for eight common food items, two for each of the following four categories: ‘high-sugar, healthy’ [one eighth of a ‘pink lady’ apple (15 g), Woolworths honey (70 g)]; ‘high-sugar, unhealthy’ [one quarter of Mars chocolate bar (14.5 g), two Pascall marshmallows (12.5 g)]; ‘high-fat, healthy’ [Woolworths olive oil (35 g), 50 mL Dairy Farmers full-fat yogurt (52 g)]; and ‘high-fat, unhealthy’ [one third of Woolworths small croissant (23 g), three Cheezels^®^ (5.5 g)]. The stimuli categorisation was based on research indicating that those with AN prefer highly sweet stimuli [34], while disliking highly fatty stimuli [31], but that hedonistic responses in ED individuals may vary based on perceived caloric value and ‘health’ status [41].

The stimuli were presented in transparent plastic jars with screw-top lids. Participants were presented with each stimulus approximately 2 cm in front of their nostrils and asked to sniff. After removal of the jar, participants rated the intensity and pleasantness of the odour. This procedure was repeated for all eight food items and presented in a randomized order for each participant. Ratings were made on a 120 mm labelled magnitude scale (LMS). Sensitivity was measured via the intensity rating which had six anchors ranging from 0 mm = barely detectable to 120 mm = strongest imaginable [42,43]. Note that the LMSs are usually 100 mm in length [42] but we used a scale of 120 mm as per our previous study [43]. The pleasantness rating had 11 anchors ranging from 0 mm = most disliked sensation imaginable to 120 = most liked sensation imaginable [43,43]. Ratings were calculated by measuring the length in mm from the bottom of the scale to the mark participants made on the LMS. A total of eight variables were created from this measure by taking the average intensity and pleasantness rating for the two stimuli within each of the four categories (i.e., high sugar/healthy, high sugar/unhealthy, high fat/healthy, high fat/unhealthy).

#### 2.2.6. Liquid Tastant Rating Task

Taste sensitivity and preference were measured using 12 different liquids consisting of three tastant types (i.e., sweet, bitter, and fatty) at four different concentrations (very low, low, medium, and high). Sucrose (CSR) was used to produce a sweet taste, caffeine (Sigma-Aldrich, Sydney, Australia) was used to produce a bitter taste, while dairy products (Woolworths, Sydney, Australia) were used to produce a fatty taste. Concentrations for the fatty tastants were: (1) high = cream; (2) medium = equal parts cream and full cream milk; (3) low = full cream milk; (4) very low = skim milk. Concentrations for the sweet and bitter tastant stimuli are depicted below in Table 1 and based on those outlined in Prescott et al. [44].

Twenty milliliters (mL) of each liquid were presented in separate, 50 mL, plastic cups in random order. While the fatty tastants had a white appearance, both the bitter and sweet tastants were clear. Participants poured the entire contents of each cup into their mouths and made five LMS ratings on a single sheet of paper: intensity; sweetness; bitterness; fattiness; pleasantness. After each rating was made, participants expectorated the stimulus, rinsed their mouth with water and then expectorated the water. Participants made a total of 60 ratings during this task because there were three types of stimuli (i.e., sweet, bitter, fatty) presented at four different concentrations (i.e., very low, low, medium, high) which were rated on five dimensions (i.e., intensity, sweetness, bitterness, fattiness, pleasantness). Therefore, a total of 60 variables were created for this measure where higher scores indicate stronger endorsement of the dimension rated.

### 2.3. Procedure

All testing took place on the university campus. Participants were instructed not to consume any food or beverages for at least one hour prior to arrival. Upon arrival, participants were provided with information and consent forms and informed consent was obtained before beginning the study. Height and weight data were recorded to obtain a BMI score. The tasks were then presented in the following order: hunger rating 1, olfactory threshold test, health interview, olfactory discrimination test, hunger rating 2, EAT-26, food odour rating task, hunger rating 3, liquid tastant rating task, hunger rating 4. The study ran for approximately 60 min in total.

### 2.4. Data Analyses

All statistical analyses were conducted using Statistical Packages for Social Sciences (version 28) [45]. Hypotheses were tested using Spearman’s rank-order correlations, as the assumption of normality was violated. Exploratory analyses were conducted to determine whether participants with low versus high EAT-26 scores (determined via a median split) rated the food stimuli and taste stimuli differently via a multivariate analysis of variance (MANOVA) and three repeated measures analysis of variance (RM-ANOVAs). Preliminary analyses of the correlations between EAT-26 scores and the taste stimuli ratings across the four concentrations revealed non-significant correlations. Moreover, we found no significant differences between the correlations across the four concentration levels. Therefore, to provide more parsimonious results, we created a mean score for each tastant type based on the ratings across the four tastant concentrations, resulting in a total of 15 variables for the liquid tastant ratings task.

Based on the initial, planned findings, exploratory analyses were conducted to explore whether any complex relationships existed between the different psychophysical ratings and eating attitudes, we categorised participants based on a median split of EAT-26 scores and conducted three analyses of variance. The median split resulted in 43 participants having lower EAT-26 scores (i.e., 5 or lower) and 37 participants with higher EAT-26 scores (i.e., 6 or higher) and the variable was named “EAT median group”. The first analysis was a 2 EAT median group (lower, higher) × 3 DVs (BMI, odour threshold, odour discrimination) ANOVA to determine whether groups different on these three dependent variables. The second analysis conducted was a 2 EAT median group (lower, higher) × 2 food type (sweet, fatty) × 2 food health (healthy, unhealthy) × 2 rating (intensity, pleasantness) repeated measures analysis of variance (RM-ANOVA) to determine whether the two groups gave different psychophysical ratings to the different food stimuli. The third analysis conducted was a 2 EAT median group (lower, higher) × 3 liquid tastant type (sweet, bitter, fatty) × 2 rating (intensity, pleasantness) repeated measures analysis of variance (RM-ANOVA) to determine whether the two groups gave different psychophysical ratings to the different taste stimuli. Note that only the liquid taste type relevant psychophysical rating (i.e., sweetness, bitterness, fattiness) plus intensity and pleasantness ratings were included in the analyses to simplify the interpretation of the findings and because intensity and pleasantness are the most commonly psychophysical attributes of interest in chemosensory research.

## 3. Results

The results are organised into four sections: assumption testing, descriptive statistics, bivariate correlations, and exploratory analyses. The final sample consisted of 80 female participants. The EAT-26 variable is continuous throughout assumption testing but is transformed into a categorical variable for the purpose of exploratory analyses. Hunger and BMI were included as potential covariates across all hypothesis tests.

### 3.1. Assumption Testing

The assumption of independence of observations was met by the study’s design, and all data were numeric. However, the assumption of normality was violated as EAT-26 scores were found to be positively skewed and mesokurtic, further supported by a significant Shapiro–Wilk test result (z = 6.17, *p* < 0.001). As a result, all hypotheses were tested using non-parametric Spearman’s rank-order correlations. All other variables of interest were approximately normally distributed.

### 3.2. Descriptive Statistics and Correlations

Table 2 displays the descriptive statistics for—and correlations between—EAT-26, BMI, hunger and olfactory ability. Table 3 displays the descriptive statistics for—and correlations between—EAT-26, BMI, olfactory ability and food odour ratings. Finally, Table 4 displays the descriptive statistics for—and correlations between—EAT-26, BMI, olfactory ability and liquid tastant ratings.

#### 3.2.1. Hypothesis 1: Were Higher EAT-26 Scores Associated with Lower Olfactory Ability?

As can be seen in Table 2, there were no statistically significant correlations between EAT-26 scores and olfactory ability (that is, odour threshold and odour discrimination scores). The only significant correlation indicated higher a BMI was associated with a higher odour discrimination score.

#### 3.2.2. Hypothesis 2: Were Higher EAT-26 Scores Associated with Higher Perceived Pleasantness of High Sugar, Healthy Food Smells?

There were no significant correlations between EAT-26 scores and any psychophysical ratings (i.e., intensity and pleasantness) for the four food odour types (i.e., high sugar, healthy; high sugar, unhealthy; high fat, healthy; high fat, unhealthy). In fact, there were no significant correlations between any food odour rating variables and the BMI, hunger total or olfactory ability.

#### 3.2.3. Hypothesis 3: Were Higher EAT-26 Scores Associated with Lower Psychophysical Ratings of Taste Stimuli?

There were no significant correlations between EAT-26 scores and psychophysical ratings (i.e., sweetness, bitterness, fattiness, intensity) for the three liquid tastant types (i.e., sweet, bitter, fatty). While all the correlations between EAT-26 and pleasantness ratings were negative, none were statistically significant.

#### 3.2.4. Hypothesis 4: Were Higher EAT-26 Scores Associated with Higher Perceived Pleasantness of Sweet Tastes?

There were no significant correlations between EAT-26 scores and pleasantness ratings for the three liquid tastant types (i.e., sweet, bitter, fatty). There was a significant correlation between bitter pleasantness and odour discrimination, indicating higher odour discrimination ability was associated with higher bitter pleasantness ratings. There were also two significant correlations involving the hunger mean score, indicating that higher hunger levels were associated with higher bitter pleasantness and fatty pleasantness ratings.

### 3.3. Exploratory Analyses: Did the Higher EAT-26 Group Differ from the Lower EAT-26 Group on Any Measures Employed?

Table 5 displays descriptive statistics of the study variables, based on the median split of EAT-26 scores.

#### 3.3.1. Did Lower and Higher EAT Groups Differ in Terms of BMI or Olfactory Ability?

The results of the ANOVA indicated there was no significant difference between the lower and higher EAT median groups in terms of BMI, odour thresholds and odour discrimination, all Fs < 1.

#### 3.3.2. Did Lower and Higher EAT Median Groups Rate the Food Odours Differently Depending on the Food Type?

**Figure 1:**
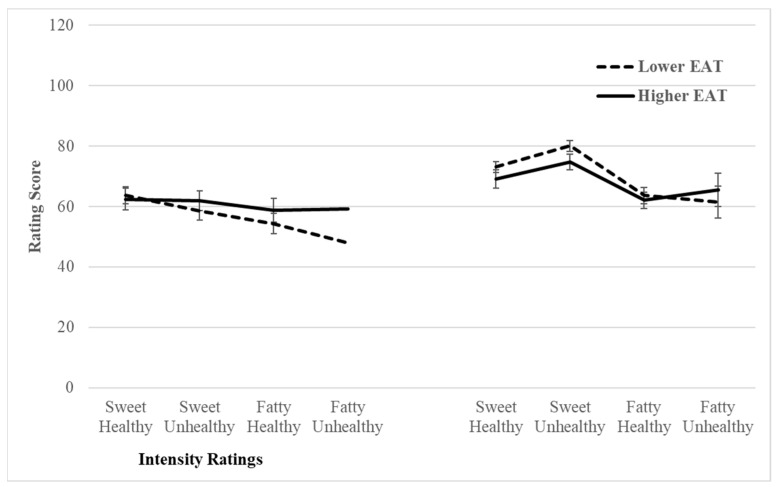
Line graphs depicting mean food odour intensity and pleasantness ratings by EAT median group.

The RM-MANOVA revealed a significant main effect for food type (i.e., sweet vs. fatty), indicating participants rated sweet and fatty food odours as smelling more intense and pleasant, *F*(1, 78) = 47.03, *p* < 0.001, $\eta_{p}^{\;}$^2^ = 0.38. Moreover, there was a significant food type (i.e., sweet vs. fatty) × EAT median group (i.e., lower vs. higher) interaction, indicating the higher group rated the odour of sweet and fatty foods as equally intense, whereas the lower group rated fatty food odours less intense than sweet food odours, *F*(1, 78) = 6.02, *p* = 0.016, $\eta_{p}^{\;}$^2^ = 0.07 (see Figure 1). Although the interaction for food health × EAT median group bordered on statistical significance (*F*(1, 78) = 3.53, *p* = 0.064, $\eta_{p}^{\;}$^2^ = 0.04), all other relevant main and interaction effects were not statistically significant.

#### 3.3.3. Did Lower and Higher EAT Median Groups Rate the Liquid Tastant Differently Depending on the Tastant Type?

The RM-MANOVA revealed a significant main effect for tastant type, indicating that participants rated sweet, bitter and fatty tastants differently in terms of intensity and pleasantness, *F*(2, 156) = 25.14, *p* < 0.001, $\eta_{p}^{\;}$^2^ = 0.24. However, the tastant type × EAT median group interaction was non-significant (*F* < 1), indicating there were no group differences in the ratings for the different tastants. All other relevant main and interaction effects were not statistically significant. Moreover, post-hoc contrast testing indicated no between group differences in terms of intensity or pleasantness ratings for each of the three tastant types.

## 4. Discussion

The aim of the present study was to determine whether higher degrees of pathological eating attitudes in a non-clinical sample are associated with smell and taste deficits and preferences. Contrary to the first hypothesis, higher levels of disordered eating attitudes were not associated with lower olfactory ability (i.e., odour threshold and odour discrimination). The second hypothesis was also not supported, as higher levels of disordered eating attitudes were not associated with higher pleasantness ratings for the odours of sweet, health foods. Similarly, neither the third or fourth hypotheses were confirmed because higher levels of disordered eating were not associated with lower psychophysical ratings of taste stimuli or higher perceived pleasantness of sweet tastes. However, exploratory between group analyses with participants classified as scoring lower or higher on the EAT-26 based on a median split, indicated that the higher group rated the odour of sweet and fatty foods as equally intense, whereas the lower group rated fatty food odours less intense than sweet food odours.

The results across all hypotheses are inconsistent with several previous findings. First, unlike our findings, previous research has found that both olfactory and gustatory ability are significantly lower in high ED populations [7,14,23] and those with AN display a preference for sweet food stimuli [26,34]. One possible reason for this discrepancy is that past findings have been produced almost exclusively within a clinical sample of AN patients, rather than across eating disorders more broadly. These discrepancies will be discussed in more detail below.

To explore the data further, EAT-26 scores were categorized via a median split. The results indicated a non-significant difference in mean BMI between the lower and higher EAT group. This finding was inconsistent with a vast body of prior research that suggests endorsement of restrictive eating is associated with caloric restriction and emaciation [46,47]. However, our finding may suggest that disordered eating attitudes are not limited to those with an underweight BMI as seen in AN. Further, this may shed light on the non-significant results of this study, as the hypotheses were developed based on research conducted almost entirely on those with AN, whereas this sample encapsulates a broader scope of disordered eaters. Therefore, future studies may benefit from broadening the inclusion of various types of disordered eating.

The median split analyses yielded a significant interaction effect which indicated that those with higher disordered eating attitudes perceive the odour of sweet and fatty foods as equally intense, whereas those with lower levels of disordered eating perceive fatty food odours less intensely than sweet food odours. While this finding may suggest differential sensitivity between those with higher and lower disordered eating attitudes in relation to the odours of sweet and fatty foods, previous studies have found no differences in gustatory sensitivity between those with an ED and the general population [26,48]. However, given the use of different participant samples and diagnostic tools, replication of our findings in future research is required.

A strength of this study was the investigation of disordered eating attitudes and behaviours beyond a clinical setting. The majority of past research has been conducted exclusively within clinical populations, specifically females with AN, thus neglecting to consider the remainder of the population where there is still likely to be high levels of disordered eating behaviours [49]. Accordingly, epidemiological data points to the conclusion that ED diagnostic criteria are overly stringent in that many individuals who fail to meet diagnostic criteria display similar psychiatric morbidity to those who are diagnosed [50].

Furthermore, past research has predominantly focused on the psychological processes behind disordered eating, which has been largely limited to self-reported measures to gain insight into the thoughts and attitudes held by individuals with EDs [51]. Thus, a strength of this study is its use of psychophysical measures to obtain information pertaining to olfactory and gustatory ability and preference beyond what self-reporting can establish. Additionally, the incorporation of real-food stimuli in these psychophysical measures enhanced the ecological validity of this study.

Another strength of this study was its targeted use of a recruitment screener to identify a sufficiently representative sample of those with disordered eating attitudes. The screener, conducted in university classes, facilitated the recruitment of participants who initially endorsed high levels of restrictive eating behaviours. As a result, nine out of 80 participants (11.25%) displayed clinical levels of disordered eating in this sample (based on EAT-26 criteria), which is greater than the approximated 1–4% incidence rate at any given time in Australian society [52].

The main limitation of this study pertains to the use of the self-report EAT-26 questionnaire. While it is a simple and economical measure of disordered eating across both clinical and non-clinical contexts, there are still concerns about social desirability bias given the sensitive nature of the self-report questions. Furthermore, while the EAT-26 is predominately utilised in people with restrictive eating behaviours, it has scarcely been utilised across other EDs more widely [53]. Finally, while it is appropriate to use the EAT-26 rather than the DSM in non-clinical samples such as ours, it is possible that different constructs of disordered eating may be captured by the two measures (i.e., EAT-26 vs. DSM) which may also account for the inconsistent findings across clinical and non-clinical studies. Accordingly, future non-clinical research would benefit from more extensive participant information to corroborate EAT-26 scores.

## 5. Conclusions

The present study aimed to investigate the association between pathological eating attitudes and both olfactory and gustatory perception and preference. This study was the first in the literature to utilise real-food stimuli to investigate this aim in a non-clinical context. While the findings of this study suggest that there may be differential sensitivity to odour of sweet and fatty foods in those with higher levels of pathological eating attitudes, the findings require replication for confirmation, especially given the non-significant associations found in the bivariate relationships. Future research may also benefit from extending the scope of disordered eating attitudes and behaviours beyond simply caloric restriction. In turn, this may promote the development of preventative and treatment strategies for individuals struggling with disordered eating, specific to the type of disorder.

## Figures and Tables

**Table 1:** Concentrations for Sweet and Bitter Tastant Stimuli.

	Concentration Level			
Tastant	Very low mM (g/L)	Low mM (g/L)	Medium mM (g/L)	High mM (g/L)
Sucrose (sweet)	20 (6.85)	40 (13.7)	145 (49.6)	420 (143.8)
Caffeine (bitter)	0.7 (0.136)	1.9 (0.369)	6.5 (1.26)	39 (7.5)

Note. mM = molarity; g/L = grams per litre.

**Table 2:** Descriptive statistics and correlations for EAT-26, BMI, hunger and olfactory ability (*n* = 80).

Variable	Descriptives		Correlations			
	Mean (SD)	Min-Max	BMI	Hunger Mean	Odour Threshold	Odour Discrim.
EAT-26 score	8.1 (8.9)	0.0–42.0	−0.05	−0.04	0.07	−0.18
Body Mass Index	22.5 (4.3)	14.5–35.1		−0.11	0.12	0.24 *
Hunger Mean	5.0 (1.9)	1.3–9.0			0.08	−0.11
Odour Threshold	6.9 (2.9)	1.0–13.0				
Odour Discrimination	9.2 (2.1)	3.0–14.0				

Note. EAT-26 = Eating Attitudes Test 26, BMI = body mass index, Discrim. = discrimination, hunger mean score = average of all four hunger ratings. * *p* < 0.05. Significant correlations are in bold font.

**Table 3:** Descriptive statistics and correlations for EAT-26, BMI, olfactory ability and food odour ratings (*n* = 80).

Food Odour Ratings	Descriptives		Correlations				
	Mean (SD)	Min-Max	EAT-26	BMI	Hunger Mean	Odour Threshold	Odour Discrim.
High Sugar, Healthy							
Odour Intensity	63.1 (21.56)	25–113	−0.11	0.06	0.04	−0.03	−0.01
Odour Pleasantness	71.3 (16.29)	29–114	−0.10	0.20	−0.01	0.13	0.12
High Sugar, Unhealthy							
Odour Intensity	60.15 (20.01)	19.5–112.5	0.02	0.02	0.03	−0.11	−0.05
Odour Pleasantness	77.65 (15.165)	31–107	−0.06	0.02	0.18	0.17	0.12
High Fat, Healthy							
Odour Intensity	56.35 (19.98)	11–107	0.10	0.07	0.12	−0.08	−0.06
Odour Pleasantness	62.9 (13.63)	32.5–95.5	−0.15	−0.10	0.08	0.16	0.11
High Fat, Unhealthy							
Odour Intensity	53.2 (23.595)	8–116.5	0.18	0.19	0.01	0.14	0.13
Odour Pleasantness	63.35 (16.985)	14–95.5	0.08	0.05	0.15	0.07	0.04

Note. EAT-26 = Eating Attitudes Test 26, BMI = body mass index, Discrim. = discrimination.

**Table 4:** Descriptive statistics and correlations for EAT-26, BMI, olfactory ability and liquid tastant ratings (*n* = 80).

Liquid Tastant Ratings	Descriptives		Correlations				
	Mean (SD)	Min-Max	EAT-26	BMI	Hunger Mean	Odour Threshold	Odour Discrim.
Sweet tastant							
Sweetness	44.9 (17.91)	1.75–97	0.00	0.04	0.05	0.21	0.13
Intensity	40.4 (15.6)	6.75–94.5	0.10	−0.02	−0.03	0.10	0.06
Pleasantness	62 (17.31)	3–116.25	−0.10	−0.08	0.09	−0.07	0.09
Bitter tastant							
Bitterness	47 (21.48)	1.75–98	0.13	−0.05	0.02	0.11	0.00
Intensity	45.5 (18.44)	2.25–91.75	0.17	0.07	−0.17	0.22 ^#^	0.01
Pleasantness	39.2 (13.37)	5–75.25	−0.18	−0.05	0.22 *	0.04	0.23 *
Fatty tastant							
Fattiness	56.8 (22.44)	5.25–111.75	0.04	0.21	−0.01	0.06	−0.06
Intensity	45.1 (19.78)	4.5–92.75	0.13	0.03	−0.02	0.01	−0.21
Pleasantness	51 (18.02)	5.75–96.25	−0.15	−0.18	0.22 *	−0.16	−0.07

Note. EAT-26 = Eating Attitudes Test 26, BMI = body mass index, Discrim. = discrimination, * *p* < 0.05. ^#^ Rounded up from 0.219, non-significant. Significant correlations are in bold font.

**Table 5:** Descriptive statistics for food odour and liquid tastant ratings by EAT median group.

Variable	Lower EAT (*n* = 43)		Higher EAT (*n* = 37)	
	Mean (SD)		Mean (SD)	
BMI	22.3 (3.66)		22.7 (4.98)	
Odour Threshold	6.8 (2.76)		7.0 (2.89)	
Odour Discrimination	9.4 (2.12)		9.0 (2.18)	
Food Category	Intensity	Pleasantness	Intensity	Pleasantness
High sugar-healthy	63.65 (20.405)	73.1 (16.83)	62.45 (23.095)	69.2 (15.6)
High sugar-unhealthy	58.65 (18.64)	80.1 (11.505)	61.85 (21.63)	74.75 (18.285)
High fat, healthy	54.35 (20.33)	63.65 (11.91)	58.7 (19.58)	62.05 (15.52)
High fat, unhealthy	48 (22.165)	61.45 (17.32)	59.2 (24.075)	65.55 (16.555)
Liquid Tastant type	Intensity	Pleasantness	Intensity	Pleasantness
Sweet mean	38.4 (13.98)	63.5 (12.1)	42.7 (17.21)	60.2 (21.93)
Bitter mean	45.0 (19.11)	40.4 (11.01)	46.2 (17.87)	37.8 (15.72)
Fatty mean	43.5 (19.37)	52.3 (14.19)	47 (20.34)	49.7 (21.78)

## Data Availability

The data collected are not available due to confidentiality reasons.
